# Aortic asprosin overexpression does not ameliorate disease pathophysiology in a murine model of Marfan syndrome

**DOI:** 10.1038/s41598-026-59187-2

**Published:** 2026-06-25

**Authors:** Prithviraj Manohar Vijaya Shetty, Andrea Matzen, Susanne Hille, Sabine Michalewski, Henrike Witthaus, Marie Noormalal, Fady Marcous, Yousef Morcos, Tarik Bozoglu, Wiebke Sommer, Gregor Warnecke, Christian Kupatt, Andreas H. Wagner, Regina Scherließ, Derk Frank, Gerhard Sengle, Oliver J. Müller, Anca Kliesow Remes

**Affiliations:** 1 Department of Internal Medicine V, University Hospital Schleswig-Holstein, University of Kiel, German Centre for Cardiovascular Research (DZHK), Partner Site North, Arnold-Heller-Str. 3, 24105 Kiel, Germany; 2 Department of Animal Welfare, CAU Kiel, Kiel, Germany; 3 Department of Internal Medicine III, University of Kiel, German Centre for Cardiovascular Research (DZHK), Partner Site North, Kiel, Germany; 4 Department of Pediatrics and Adolescent Medicine, Faculty of Medicine, University Hospital Cologne, University of Cologne, Cologne, Germany; 5 Center for Biochemistry, Faculty of Medicine, University Hospital of Cologne, Joseph-Stelzmann-Street 52, 50931 Cologne, Germany; 6 Klinik und Poliklinik für Innere Medizin I, University Clinic rechts der Isar, Technical University Munich, Munich, Germany; 7 DZHK (German Center for Cardiovascular Research), Partner Site Munich Heart Alliance, Munich, Germany; 8 Department of Cardiac Surgery, University of Kiel, German Centre for Cardiovascular Research (DZHK), Partner Site North, Kiel, Germany; 9 Department of Cardiovascular Physiology, Heidelberg University, Heidelberg, Germany; 10 Department of Pharmaceutics and Biopharmaceutics, Kiel University, Kiel, Germany; 11 Center for Molecular Medicine Cologne (CMMC), University of Cologne, Robert- Koch-Street 21, 50931 Cologne, Germany; 12 Cologne Center for Musculoskeletal Biomechanics (CCMB), 50931 Cologne, Germany; 13 Cologne Excellence Cluster on Cellular Stress Responses in Ageing-Associated Diseases (CECAD), University of Cologne, 50931 Cologne, Germany

**Keywords:** Cardiology, Cell biology, Diseases, Genetics, Molecular biology

## Abstract

Marfan syndrome (MFS) is a connective tissue disorder caused by *FBN1* mutations, characterised by aortic aneurysms leading to life-threatening dissections. Asprosin is the C-terminal propeptide of fibrillin-1 that was shown to function as a glucogenic hormone with critical implications in vascular pathology. However, its role in MFS remains unknown. Here, we investigate the effect of asprosin overexpression in vascular smooth muscle cells (VSMCs) and in the Marfan mouse model *mgR/mgR* via endothelial-targeted transduction by adeno-associated viruses (AAVs). In both human and murine VSMCs, asprosin overexpression did not alter VCAM1 or MMP9 expression, while TNF-α-stimulated asprosin-overexpressing MOVAS cells exhibited reduced IL6 levels. In *mgR/mgR* mice, asprosin overexpression did not affect aortic diameter or elastin integrity. Molecular analyses revealed no significant change in inflammatory or epithelial-to-mesenchymal transition (EMT) markers at the mRNA or protein level, with VCAM1 and MMP9 remaining unchanged. Together, these findings indicate that asprosin overexpression does not ameliorate the aneurysmal phenotype in MFS.

**Supplementary Information:**

The online version contains supplementary material available at 10.1038/s41598-026-59187-2.

## Introduction

Marfan syndrome (MFS) is a hereditary multisystem connective tissue disorder with manifestations in the pulmonary, nervous, cardiovascular, and ocular systems^1^. Affecting roughly 1 in 5,000 individuals, patients present with dural ectasia, dolichostenomelia, spontaneous pneumothorax, aortic aneurysms, and life-threatening aortic dissection^1,2^. Of all the forms of manifestations, progressive aortic root dilation and aneurysms are the most significant cause of mortality and morbidity, emphasizing the need for understanding the molecular basis of MFS vasculopathy.

In 1991, three independent studies identified mutations in the *FBN1* gene as the underlying cause of MFS^3,4^. Located on chromosome 15q21.1, *FBN1* encodes a 350-kDa extracellular glycoprotein that is an essential structural component of microfibrils^5^. These microfibrils are integral to connective tissues, where they provide elasticity and tensile strength to the skin, ligaments, heart valves, aorta, and other organs^6^. To date, more than 3,000 distinct *FBN1* mutations have been reported in association with MFS. A single-center cohort study spanning 26 years alone identified 713 *FBN1* variants in 934 probands, underscoring the extraordinary genetic heterogeneity of the disorder^7^. While the pathophysiology of MFS has traditionally been thought to be a result of the extracellular matrix defect, recent data suggest that the signalling pathway downstream to abnormal fibrillin-1 significantly contributes to the disease progression.

Beyond its structural role in the connective tissue system, fibrillin-1 also serves as the precursor of asprosin, an orexigenic and glucogenic hormone^8^. First described in 2016, asprosin is a 140 amino acid peptide generated by furin-mediated cleavage of profibrillin-1^8^. Initially reported to be secreted by white adipose tissue during fasting^9^, subsequent studies have demonstrated asprosin expression in pancreatic β-cells^10^, cardiac^11^, and skeletal muscle, cartilage^12^, liver^11^, and other tissues, supporting the view that asprosin functions as a multi-tissue hormone.

On a molecular level, asprosin binds to the olfactory receptor G protein-coupled receptor (OLFR734) in hepatocytes, promoting glucose release via activation of the cyclic adenosine monophosphate-protein kinase A (cAMP-PKA) pathway^13^. Furthermore, asprosin stimulates appetite by binding to the protein tyrosine phosphatase receptor δ (PTPRD) in hypothalamic agouti-related protein (AgRP) neurons, highlighting that asprosin mediates its action through receptor and tissue-specific mechanisms^14^. Additionally, elevated asprosin levels have been linked to metabolic diseases like type 2 diabetes, cardiovascular disease, and polycystic ovarian syndrome^15,16^, conditions characterised by vascular inflammation and endothelial dysfunction.

In recent years, asprosin has been proven to be linked with vascular inflammation and endothelial dysfunction^17^. However, the contribution of asprosin to the vasculopathy of a multisystemic connective tissue disease like MFS remains an open question. Given that asprosin is a direct derivative of fibrillin-1, mutations in FBN1 may alter production or signalling of asprosin, in turn influencing vascular homeostasis. However, the link between asprosin and the vasculopathy in MFS has not been previously explored.

The objective of this study is to evaluate the contribution of asprosin in the vascular pathogenesis associated with MFS. We hypothesized that FBN1 mutations alter asprosin protein levels, leading to an abnormal asprosin signalling cascade, further promoting vascular dysfunction and aortic aneurysm formation. Accordingly, restoration of asprosin signalling may mitigate aortic wall damage. To test this hypothesis, we overexpressed asprosin in *mgR/mgR* mice using AAV packaged in SLRSPPS-nanoparticles to enhance endothelial homing^18^; however, the CMV promoter enabled transgene expression across the aortic wall. Because the mgr mouse models fibrillin-1 loss-of-function, asprosin overexpression provides a strategy to test whether augmenting a downstream effector can modify Marfan syndrome pathology. Parallelly, we overexpressed asprosin in primary human aortic VSMCs and Mouse vascular aortic SMCs (MOVAS) cells, to assess its effects on markers of phenotypic modulation and inflammation. Because inflammation is a key driver of aneurysm progression, hVSMCs were additionally stimulated with tumor necrosis factor-alpha (TNF-α), a well-established inducer of pro-inflammatory responses in vascular smooth muscle cells. Through these in vitro and in vivo approaches, we aim to determine whether restoration of asprosin activity can rescue MFS-associated vasculopathy.

## Results

### Asprosin influences phenotypic but not inflammatory markers in TNF-α-stimulated VSMCs

**Fig. 1:**
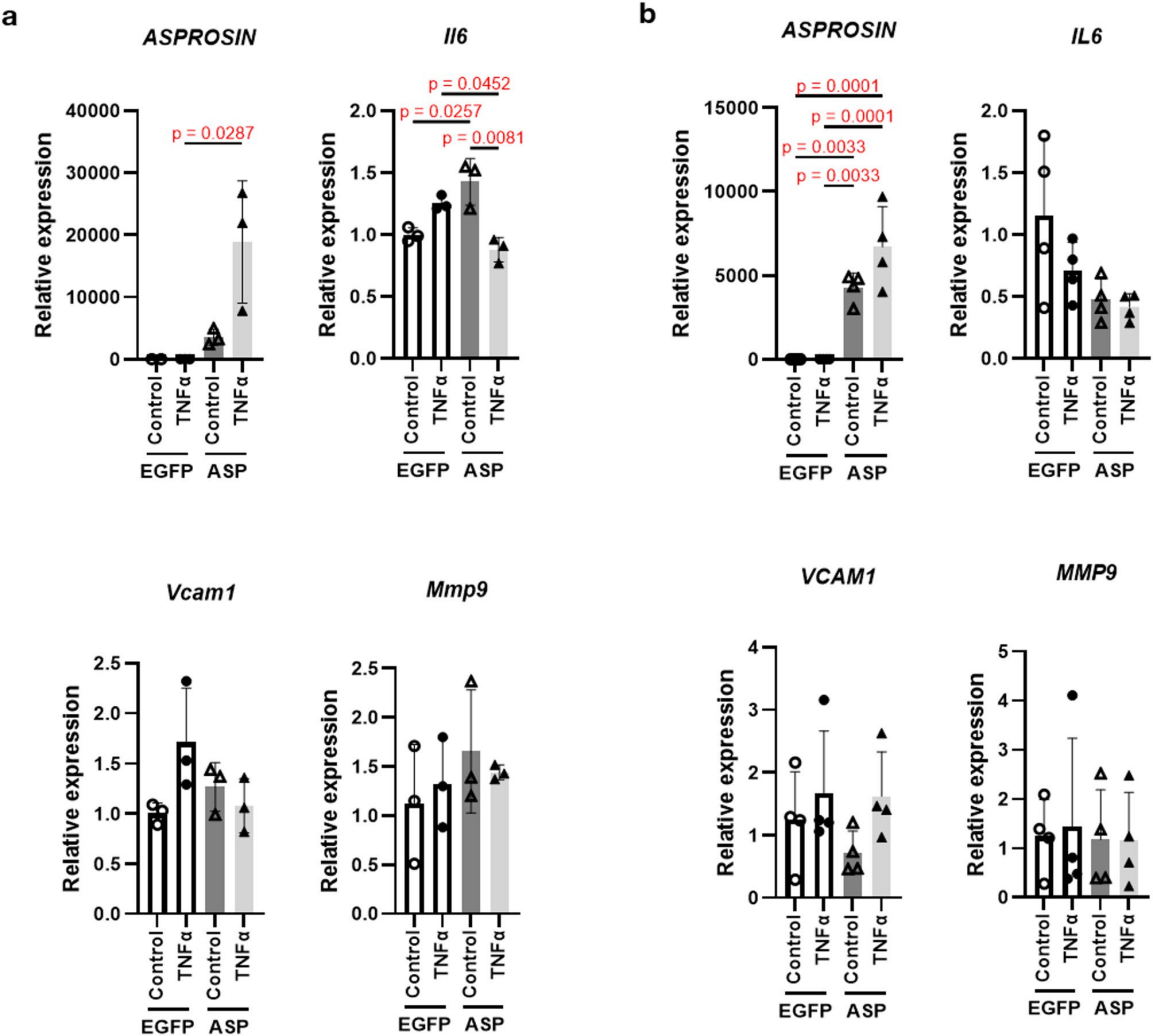
Effects of asprosin overexpression on pro-inflammatory and phenotypic markers in VSMCs. **a** Statistical quantification of mRNA levels of asprosin, *Il6*, *Vcam1*, and *Mmp9* in MOVAS cells transduced with AAV9SLR-EGFP or AAV9SLR-asprosin and stimulated with or without TNF-ɑ. **b** Statistical quantification of the same mRNA targets in primary hVSMCs under similar experimental conditions. Data are represented as mean ± SEM.

To confirm functional activity of the transgene, HUVECS were transduced with either AAV9-EGFP or AAV9-asprosin. AAV9-asprosin-transduced cells produced significantly more intracellular cAMP, supporting previously reported asprosin signaling^19^. Given the established role of inflammation in the MFS pathology^20,21^, we first examined the effect of asprosin overexpression in TNF-α-stimulated MOVAS and primary hVSMCs, as VSMCs are the primary effectors of extracellular matrix maintenance and aneurysm pathogenesis. Both cell types showed positive asprosin transgene expression, in line with our previous studies in which the AAV9SLR vector achieved ~ 90% transduction efficiency^22^. As shown in Fig. 1a, b, overexpression of asprosin in both murine and primary human VSMCs did not significantly alter VCAM1 mRNA levels under basal conditions or upon TNF-α exposure. In MOVAS cells, asprosin transduction led to a modest (~ 1.4-fold) increase in *Il6* levels compared to EGFP controls. In contrast, TNF-α stimulation in the presence of asprosin led to a significant decrease in control-treated cells. Expression of MMP9 remained unchanged across all experimental conditions (Fig. 1a, b). pSMAD3 proteins levels showed a significant increase in asprosin transduced MOVAS, but TNF-α stimulation showed no difference (Supplementary Figure S3).

### Aortic asprosin overexpression does not affect aortic pathology in Marfan mice

**Fig. 2:**
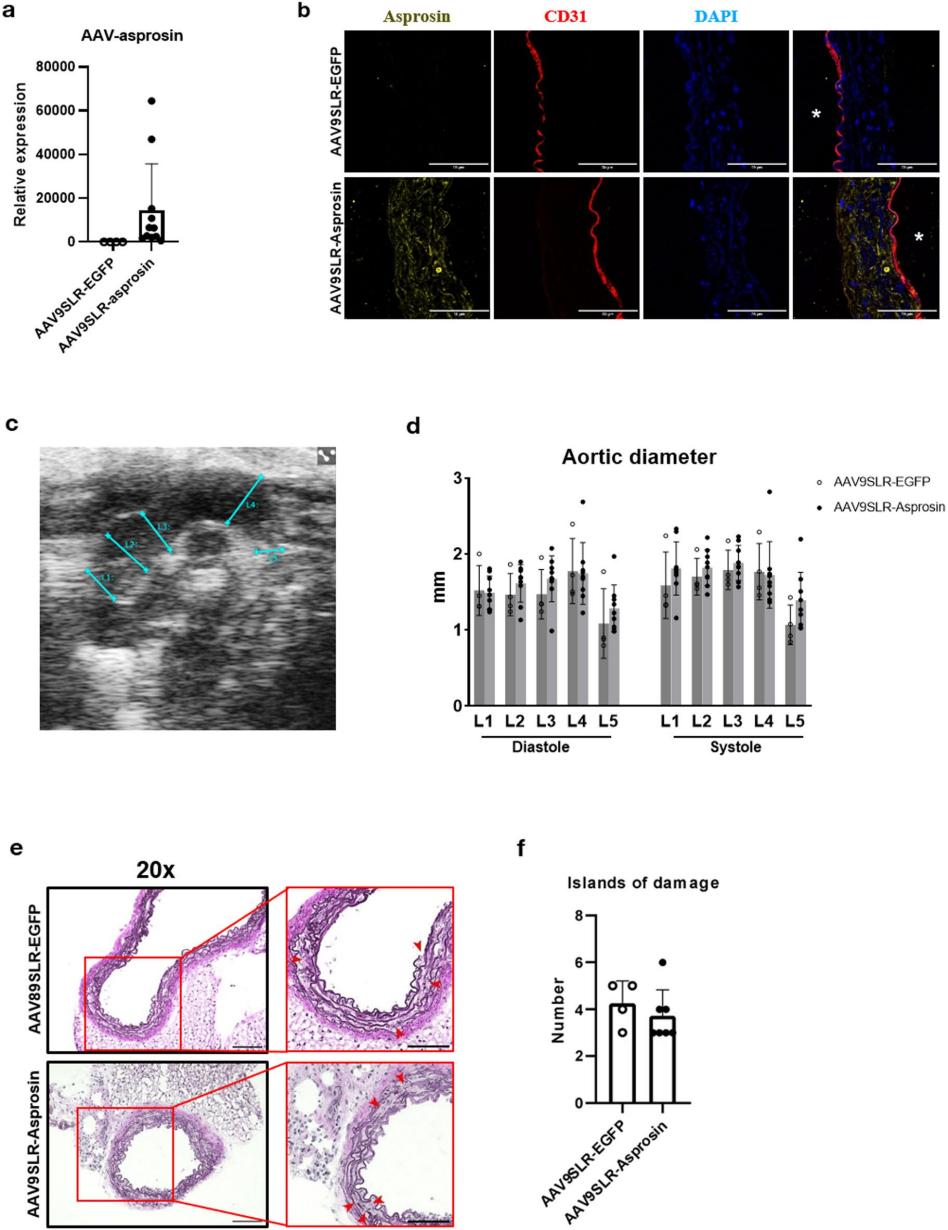
Aortic asprosin overexpression and structural assessment in aortae of *mgR/mgR* mice. **a** qPCR analysis of asprosin transgene in the thoracic aorta of AAV9SLR-EGFP or AAV9SLR-asprosin injected *mgR/mgR* mice. **b** Immunohistochemical staining of asprosin and CD31 in thoracic aortic tissues. Scale bar: 50 μm. **c** Transthoracic echocardiographic image of the aorta indicating the five standard measurement sites: aortic annulus (L1), sinuses of Valsalva (L2), sinotubular junction (L3), aortic arch (L4), and proximal descending aorta (L5). **d** Histogram showing systolic and diastolic aortic diameters measured at the five standard sites. **e** Representative images of thoracic aortic section stained with Van Gieson; red arrows indicate islands of damage. Scale bar: 100 μm (x 20) and 50 μm (magnified images). **f** Quantification of islands of damage. Scale bar: 100 μm. Data are represented as mean ± SEM; For AAV9SLR-EGFP, *n* = 4; AAV9SLR-asprosin, *n* = 11.

Consistent with previous reports^23^, female *mgR/mgR* mice exhibited improved survival compared to males (Supplementary Figure S1), supporting their use for subsequent experiments. To assess the in vivo impact of asprosin, we achieved endothelial cell-specific overexpression by injection of AAV9-EGFP or AAV9-asprosin driven by the CMV promoter fused with an endothelial-affine peptide^18,24^ in *mgR/mgR* mice. No significant difference between weights of mice were observed (AAV9-EGFP = 23.25 ± 1.13; AAV9-asprosin = 22.08 ± 3.63). As depicted in Fig. 2a, we detected robust transgene overexpression in the aortic tissue of AAV9-asprosin-treated mice. Consistent with the transcript data, asprosin immunostaining confirmed asprosin localization within the aortic wall on the protein level (Fig. 2b). Despite successful transduction, the aortic diameter of the asprosin-overexpressing mice was comparable to AAV9-EGFP-injected controls (Fig. 2c, d). Furthermore, histological assessment using Van Gieson staining revealed no differences in the number of islands of damage between the two groups, suggesting no effect on elastin integrity (Fig. 2e, f).

### Aortic asprosin overexpression does not affect inflammatory or epithelial-to-mesenchymal transition (EMT) markers

**Fig. 3:**
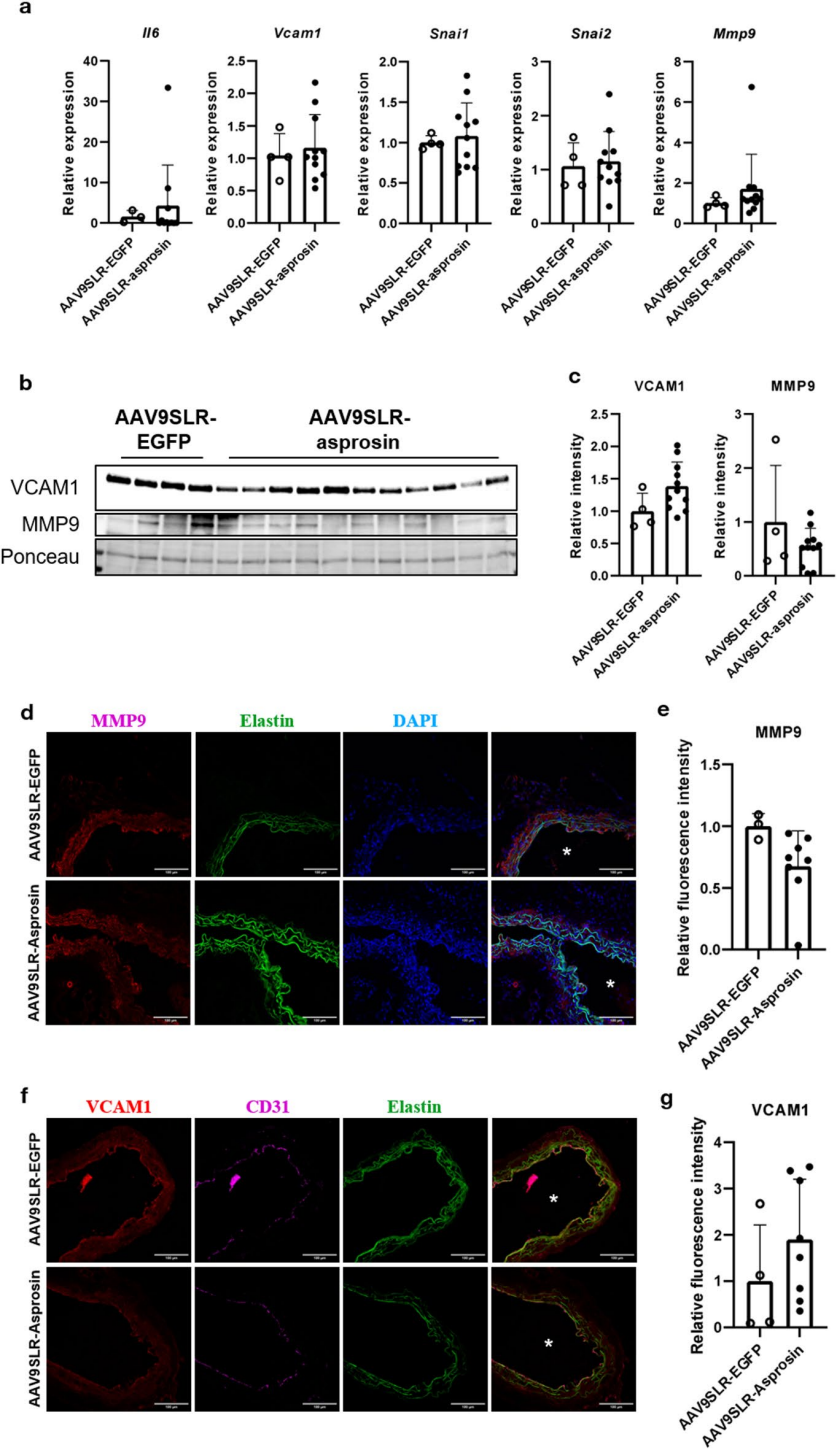
Effect of aortic overexpression of asprosin on inflammatory and phenotypic markers in the aorta of *mgR/mgR* mice. **a** Histograms representing mRNA levels of Il6, Vcam1, Snai1, Snai2, and Mmp9 in the thoracic aorta of *mgR/mgR* mice injected with AAV9-EGFP or AAV9-asprosin. **b** Representative western blot images and **c** corresponding quantification of VCAM1 and MMP9 protein levels in aortic tissues. **d** and **f** Immunohistochemical staining and **e** and **g** corresponding quantification of MMP9 and VCAM1 in thoracic aortic tissues. Scale bar: 100 μm. Data are represented as mean ± SEM; For AAV9SLR-EGFP, *n* = 4; AAV9SLR-asprosin, *n* = 11.

We next aimed to explore further molecular changes associated with asprosin overexpression in *mgR/mgR* mice. As shown in Fig. 3a, expression of inflammatory genes *Vcam1* and *Il6* was comparable between the two experimental groups. EMT-related genes (*Snai1* and *Snai2*) and matrix metalloproteinase *Mmp9* likewise showed no significant difference on the transcript level (Fig. 3a). Consistently, Western blot analysis revealed no significant change in VCAM1 or MMP9 protein levels following asprosin overexpression (Fig. 3b, c). These results were further supported by immunohistochemical staining of aortic sections for MMP9 and VCAM1, which showed no differences across conditions (Fig. 3d–g).

## Discussion

Here, we set out to determine whether asprosin, the C-terminal fibrillin-1 propeptide, influences the vascular manifestations of MFS. Importantly, we report for the first time the successful aortic overexpression of asprosin in the Marfan mouse model *mgR/mgR*. Despite its dependence on fibrillin-1 and reported involvement in metabolic and vascular diseases, our results indicate that aortic overexpression of the hormone asprosin did not alter aortic diameter, elastin integrity, or pro-inflammatory or EMT markers.

MFS is a connective tissue disorder characterized by aortic aneurysm and dissection due to the weakening of the ECM caused by fibrillin-1 defects. Asprosin was reported to be associated with various metabolic and vascular diseases, such as obesity, type 2 diabetes, hypertension, and atherosclerosis^25–27^. However, our study shows that AAV-mediated supplementation of asprosin does not rescue vascular defects in Marfan mice. This disparity may arise from the complexity of MFS pathophysiology, in which factors beyond asprosin may drive the vascular phenotype.

Several recent studies have established asprosin as a potent mediator of vascular pathology. Studies on spontaneously hypertensive rats showed elevated asprosin levels in VSMCs isolated from the aortic media^28^. Moreover, asprosin overexpression in VSMCs promoted oxidative stress, migration, and proliferation via NOX1/2 upregulation^28^. Increased asprosin levels have also been shown to exacerbate VSMC remodeling and neointima formation. Interestingly, in atherosclerosis models, asprosin treatment increased plaque area, thereby supporting disease progression^26^. Conversely, one study reported that asprosin prevents lipid accumulation in macrophages and lowers the atherosclerosis burden in apoE−/− mice^27^. Our study extends this body of work by exploring asprosin function in a hereditary connective tissue disease such as MFS, in which vascular pathology arises from intrinsic ECM weakness rather than acquired metabolic stress. To mimic inflammatory stress in vascular smooth muscle cells, TNF-α has been employed as a strong inflammatory mediator of vascular inflammation. VSMCs express TNFR1 constitutively, and TNF-α activation of NF-κB and MAPK pathways leads to cytokine production and a synthetic inflammatory phenotype relevant to vascular remodeling during aneurysm formation^29–31^.

Contrary to our study, asprosin was found to promote NLRP3 inflammasome activation in VSMCs^32^ and to drive endothelial cell inflammation by activating the NF-κB pathway^17^. Importantly, asprosin inhibition has been shown to alleviate endothelial dysfunction and vascular phenotypic shift, highlighting its potential as a therapeutic target^33^. The lack of major phenotypic effects here implies that the vascular actions of asprosin may depend on disease context, receptor availability, and interaction with specific pro-inflammatory pathways. Disease-specific fibrillin-1 damage and downstream signaling cascades in MFS may overshadow, if not negate, the impact of asprosin signaling. Hence, in contrast to secondary vasculopathies, in which asprosin may exacerbate or ameliorate vascular pathophysiology, overexpression is apparently neither protective nor deleterious in hereditary matrix disorders like MFS.

Given the severe aortic phenotype of male *mgR/mgR* mice, further studies may reveal the effects of asprosin under heightened vascular stress, presumably overcoming the temporal limitations of this study. Another limitation is the low resolution of the ultrasound images, restricting the detection of subtle changes in the murine aorta. Nevertheless, the absence of consistent differences across measurements is in line with other findings, suggesting lack of remodeling within the 4-week period. Additionally, further experiments could explore the inhibition of asprosin, as it may reveal whether its role in MFS aligns with reports of protective effects in other models^16^. Moreover, in vitro studies could include the use of other inflammatory stimuli like IFN-γ, LPS, and particularly TGF-β to understand diverse signaling cascades in the aortic wall. This is relevant given the emerging view that Marfan vasculopathy is driven by multiple interacting signalling cascades rather than a single dominant pathway. Recent works support a multifactorial model in which extracellular matrix dysfunction, VSMC dysfunction, and immune-mediated inflammation contribute in parallel. For example, inhibition of CX3CR1 + aortic macrophages is seen to alleviate TAA progression in Marfan mice^34^, highlighting a macrophage-driven inflammatory angle. At the same time, other studies underline roles of mechanosensing defects, oxidative stress, inflammation, and VSMC phenotypic switching beyond canonical TGF-β signaling^35,36^. Together, these observations suggest that experimental approaches incorporating various inflammatory stimuli may better capture the complex pathophysiology underlying Marfan vasculopathy.

Studying other cell types like endothelial cells, fibroblasts, or pericytes may reveal cell-specific responses to asprosin. Finally, to model the intercellular interactions within the vascular microenvironment, co-culture approaches can be employed.

Although the absence of overt structural or inflammatory changes may initially appear negative, our results offer valuable insights into the specificity of the biological functions of asprosin. MFS is primarily a disorder of the ECM, in which fibrillin-1 mutations drive aortic aneurysm formation through defective microfibril architecture and dysregulated TGF-β signalling^37^. Our findings suggest that, despite being derived from the same precursor, asprosin does not independently contribute to ECM remodelling in this context. This distinction provides a significant step forward in understanding how fibrillin-1 derived fragments diverge in function, with some fragments modulating cell signalling^38,39^ and others, such as asprosin, maintaining roles primarily outside the structural matrix environment.

Notably, the demonstration that asprosin can be efficiently overexpressed in the aorta using SLRSPPS peptide-coated AAV9 vectors represents a methodological advance. This approach establishes a versatile platform to study secreted hormones and peptides directly within the vascular wall, enabling future investigations into asprosin signalling under physiological and pathological conditions. Moreover, the observation that asprosin did not exacerbate aortic dilatation or elastin fragmentation supports its safety in vascular disease, an essential consideration for ongoing translational studies targeting asprosin in metabolic diseases.

In summary, we report the first targeted overexpression of asprosin in the aorta and show that, under the conditions tested, it does not impact the molecular or structural features of aortic disease in MFS. Although asprosin is derived from fibrillin-1 and has been linked to metabolic and inflammatory signaling, its overexpression did not influence VCAM1, MMP9, or other remodeling markers in our cell and mouse models. These findings redefine our understanding of asprosin biology by demonstrating its limited role in genetically-driven ECM disorders. Additionally, the methodological framework established here provides a foundation for future studies exploring asprosin signaling in both inherited and acquired vascular disorders.

## Materials and methods

### AAV production

AAV9SLR and AAV9, with cytomegalovirus (CMV) promoter driving the EGFP or asprosin cDNA, were produced and further purified as described earlier^40,41^. For endothelial transduction in vivo, AAV9 was coated with endothelial-affine peptide as described previously^18^. In brief, AAV9 and SLRSPPS- nanoparticles were incubated in Opti-MEM for 30 min to form complexes before injection into mice. For in vitro transductions, AAV9SLR virus was used^38^.

### Animal study

All animal procedures were approved by the Ministry of Energy Transition, Agriculture, Environment, Nature and Digitalization of Schleswig-Holstein, Germany (permission no. V242-55381/2019). All animals were bred in-house at the Zentrale Tierhaltung (ZTH), UKSH, Kiel. All experiments were performed in accordance with the German Animal Welfare Act (TierSchG) and Directive 2010/63/EU of the European Parliament on the protection of animals used for scientific purposes. The study is reported in accordance with the ARRIVE guidelines. Female *mgR/mgR* mice aged 8–10 weeks old were used for this study as male mgr mice display aggressive disease phenotype with premature mortality^42^.This age range gives enough time for AAV-administration and monitoring of respective transduction effects. Mice were housed with *ad libitum* feeding and 10 h/14 h light/dark cycles. For endothelial overexpression in vivo, AAV9 with the EGFP or asprosin cDNA under control of the CMV promoter was coated with the endothelial-affine SLRSPPS peptide before injection into mice. A total volume of 100 µL of 10^12^ viruses was injected in the tail vein of the mice. Four weeks after injection, aortic diameters were measured. Mice were then euthanized by cervical dislocation, and the thoracic aorta (and blood samples) were collected for subsequent analyses. Post sacrifice, aortae were isolated, washed with PBS, and divided: the top part for imaging, the middle for protein isolation, and the bottom for RNA isolation.

### Aortic diameter assessment

Aortic diameters were assessed using the Vevo 1100 ultrasound imaging system (VisualSonics, Fujifilm Sonosite B.V.) equipped with an MS400 transducer (30 MHz central frequency, 7.0 mm focal length). Mice were anesthetized in an induction chamber with 3% isoflurane in 100% oxygen at 1 L/min for 1–2 min, then maintenance with 1.5-2% isoflurane delivered via a nose mask^43^. Following anesthesia, aortic diameters were measured at four specific points, namely the aortic annulus (L1), sinuses of Valsalva (L2), sinotubular junction (L3), aortic arch (L4), and proximal descending aorta (L5), as previously described^43^.

### Isolation of primary human VSMCs

Aortic tissues were collected from patients undergoing surgery at the University Hospital Schleswig-Holstein, Kiel, Germany (permission no. D495/19 by the Ethics Committee of the University Medical Centre Schleswig-Holstein, Kiel). All procedures complied with the Declaration of Helsinki & relevant institutional guidelines and regulations. Human VSMCs were isolated as previously described^44^. Briefly, aortae were washed with sterile PBS, and the tunica media was isolated and chopped into fine pieces. The tissue was then enzymatically digested using 0.1% collagenase type 1 (Worthington, Columbus, OH, USA) at 37 °C for 3 h. The resultant solution was filtered and cultured at 37 °C, 5% CO_2_. Cultured cells were used for up to 10 passages for subsequent experiments.

### AAV transduction and TNF-ɑ treatment of hVSMCs and MOVAS

MOVAS cells (mouse aortic vascular smooth muscle cells; Mus musculus, strain C57BL/6 mice) were obtained from ATCC (catalog number CRL-2797). Cells were used between passages P3 and P8. Cells were used within a restricted passage range and maintained under standardized culture conditions to limit phenotypic drift. Cells were cultured in DMEM (Pan-biotech, P04-03600) supplemented with 10% fetal bovine serum, L-Glutamine (Gibco, 25030), and Pen Strep (Gibco, 15140). Both hVSMCs and MOVAS were plated in cell culture dishes and cultured untill 90% confluency. Cells were then transduced with AAV9SLR vectors encoding either EGFP or asprosin and incubated for 48 h. Subsequently, these cells were stimulated with 1 ng/mL TNF-ɑ and incubated for an additional 24 h before harvesting for RNA isolation.

### qRT-PCR

Total RNA was extracted from aortic tissues using RNeasy kit (Qiagen) and from hVSMC and MOVAS cells using Quick-RNA Miniprep kit (Zymo Research) according to the manufacturer’s instructions. cDNA was synthesized using LunaScript RT SuperMix Kit (New England Biolabs). qRT-PCR was performed using specific gene primers (Supplementary Table S1) and SYBR Green (Qiagen). Gene expression levels were normalised to Ribosomal protein L32 (*Rpl32*) for mouse samples and to Ribosomal protein lateral stalk subunit P0 (*RPL0*) for human samples.

### Western blot analysis

Western Blot analysis was performed according to a standard protocol. Briefly, proteins isolated using radioimmunoprecipitation assay (RIPA) lysis buffer with protease inhibitors were electrophoresed in 10% SDS-PAGE and blotted on nitrocellulose or a polyvinylidene fluoride membrane. After blocking with 5% nonfat milk or 5% BSA, membranes were incubated overnight with primary antibodies at 4 °C. The primary antibodies used are as follows: MMP9 (Abcam, ab38898, 1:1,000), VCAM1 antibody (Abcam, ab134047, 1:1,000), β-actin (Sigma-Aldrich, A5441, 1:10,000), phospho-SMAD2 (Cell Signalling Technology, #3108, 1:1,000), phospho-SMAD3 (Cell Signalling Technology, #9520, 1:1,000), SMAD2/3 (Cell Signalling Technology, #5678, 1:1,000) and ɑSMA (Abcam, ab20509, 1:1,000). Total protein stained with Ponceau S solution (Sigma-Aldrich, P7170) served as controls, proving equal loading of protein samples. Further corresponding Horse radish peroxidase (HRP) tagged secondary antibodies (anti-rabbit HRP and anti-mouse HRP) were incubated for 1 h for detection. Imaging was performed with the ChemiDoc MP Imaging system (Bio-Rad Laboratories, Inc.).

### Immunohistochemistry

Aortic pieces were embedded in Tissue-Tek (Weckert Labortechnik) and snap frozen in liquid nitrogen-cooled isopentane. Transverse sections (7 μm thickness) were cut using a Cryostat (Microm HM 500 O). For each animal six slides containing three serial sections per slide were prepared. Sections were stored at -80 °C before staining. Aortic sections were fixed with 4% paraformaldehyde for 10 min, followed by blocking for an hour with 2% BSA containing 0.02% Triton X-100. Primary antibodies against asprosin^12^, MMP9 (Abcam, ab38898, 1:1,000), VCAM1 (Abcam, ab134047, 1:1,000), and CD31 (Santa Cruz, sc-181916, 1:200) were diluted in 1% BSA and incubated overnight at 4 °C. Respective secondary antibodies, donkey anti-rabbit Alexa Fluor 546 (Invitrogen, A10040, 1:250), chicken anti-rat Alexa Fluor 647 (Invitrogen, A21472, 1:250) and donkey anti-rabbit Alexa 568 (#A10042, Invitrogen, 1:300), were incubated for 1 h at room temperature along with DAPI (1:1,000) for nuclear staining. Fluorescence imaging was performed using a confocal microscope (LSM800, Zeiss, Oberkochen, Germany). Images were acquired using a 20x objective, at a resolution of 1024 × 1024 pixels, and detector settings were kept constant across all samples. Gain and offset were maintained identically for all groups within each experiment. For quantification, the entire section was defined as ROI (Regions of Interest). For each section, 2–3 non-overlapping images were acquired to capture the full vessel circumference. Images were analyzed individually, and measurements from images corresponding to the same section were averaged to generate a single value per section. Image analysis was performed using ImageJ (Fiji version 1.51p, National Institutes of Health, Bethesda, MD, USA). The thresholding parameters were applied uniformly to all images. Quantification was conducted in a blinded manner to the experimental group. For each animal, measurements from multiple ROIs and sections were averaged to generate a single biological replicate for statistical analysis.

### Van Gieson staining

Elastin was visualized with van Gieson staining following the manufacturer’s instructions (Sigma-Aldrich) as mentioned earlier^45^, and images were captured via bright-field microscopy (Keyence). Images were acquired using a 20x objective, at a resolution of 1024 × 1024 pixels, and detector settings were kept constant across all samples. To quantify the entire vessel, we defined the full cross-section as the region of interest (ROI). We captured two to three non-overlapping images per section to ensure the entire circumference was represented. Each image was analyzed independently, and the resulting data were averaged to produce a single representative value for each vessel section. A blinded observer quantified elastin fragmentation by counting the number of damage islands per cross-section, as previously described^34,45^. A damaged island was defined as a region containing at least two fragmented elastic fibers separated by abnormal connective tissue^45^. Only clearly defined, non-overlapping regions were counted as individual islands.

### Measurement of intracellular cAMP levels

HUVECs (human umbilical vein endothelial cells; *Homo sapiens*) were obtained from PromoCell and used between passages P2 and P8. Cells were maintained under standardized culture conditions in complete endothelial cell growth medium MV2 (PromoCell, C-22121) to minimize phenotypic drift. HUVECs were transduced with either AAV9SLR-EGFP or AAV9SLR-asprosin and incubated for 72 h to allow for transgene expression. Cells were lysed, and intracellular cAMP levels were measured using a competitive immunoassay kit (Sigma-Aldrich, CA201) according to the manufacturer’s instructions. Absorbance was recorded, and cAMP concentrations were calculated based on a standard curve.

### Statistical analysis

In vitro experiments were conducted with biological replicates as specified in the corresponding figure legends, with three technical replicates per independent sample. Mice were age- and sex-matched, and analyses of ultrasound images and elastin break quantification were performed in a blinded manner. Statistical analyses were carried out using GraphPad Prism (version 9). Normality of datasets was assessed using the D’Agostino–Pearson test. Comparisons between two groups were performed using an unpaired two-tailed Student’s t-test, while analyses involving three or more groups were evaluated by two-way ANOVA followed by appropriate post hoc testing. A p-value < 0.05 was considered statistically significant, with exact p-values below this threshold indicated in the figures.

## Supplementary Information

Below is the link to the electronic supplementary material.

Supplementary Material 1

## Data Availability

The datasets used and analysed during the current study is available from the corresponding author on reasonable request.
