# Supplementary material for: Aortic asprosin overexpression does not ameliorate disease pathophysiology in a murine model of Marfan syndrome
Source: Sci Rep. 2026 Jun 25;16:19608. doi: 10.1038/s41598-026-59187-2 (PMC13303822; doi:10.1038/s41598-026-59187-2)
Supplement: Supplementary file 1 — Supplementary Material 1 [file 41598_2026_59187_MOESM1_ESM.pdf]

Aortic asprosin overexpression does not ameliorate disease pathophysiology in a murine model of Marfan syndrome

Prithviraj Manohar Vijaya Shetty[1], Andrea Matzen[2,3], Susanne Hille[1], Sabine Michalewski[1], Henrike Witthaus[1], Marie Noormalal[3], Fady Marcous[4,5], Yousef Morcos[4,5], Tarik Bozoglu[6,7], Wiebke Sommer[8], Gregor Warnecke[8], Christian Kupatt[6,7], Andreas H Wagner[9], Regina Scherließ[10], Derk Frank[3], Gerhard Sengle[4,5,11,12,13], Oliver J Müller[1,*], Anca Kliesow Remes[1,*]

[1]Department of Internal Medicine V, University of Kiel, and German Centre for Cardiovascular Research (DZHK), partner site North, Germany

[2]Department of Animal Welfare, CAU Kiel, Kiel, Germany

[3]Department of Internal Medicine III, University of Kiel, and German Centre for Cardiovascular Research (DZHK), partner site North, Germany

[4]Department of Pediatrics and Adolescent Medicine, Faculty of Medicine and University Hospital Cologne, University of Cologne, Cologne, Germany

[5]Center for Biochemistry, Faculty of Medicine, University Hospital of Cologne, Joseph-Stelzmann-Street 52, 50931 Cologne, Germany

[6]Klinik und Poliklinik für Innere Medizin I, University Clinic rechts der Isar, Technical University Munich, Germany

[7]DZHK (German Center for Cardiovascular Research), Partner Site Munich Heart Alliance, Munich, Germany

[8]Department of Cardiac Surgery, University of Kiel, and German Centre for Cardiovascular Research (DZHK), partner site North, Germany

[9]Department of Cardiovascular Physiology, Heidelberg University, Heidelberg, Germany

[10]Department of Pharmaceutics and Biopharmaceutics, Kiel University, Germany.

[11]Center for Molecular Medicine Cologne (CMMC), University of Cologne, Robert-Koch-Street 21, 50931 Cologne, Germany

[12]Cologne Center for Musculoskeletal Biomechanics (CCMB), 50931 Cologne, Germany

[13]Cologne Excellence Cluster on Cellular Stress Responses in Ageing-Associated Diseases (CECAD), University of Cologne, 50931 Cologne, Germany

[*]Corresponding author: Anca Kliesow Remes, Dept. of Internal Med. V, University Hospital Schleswig-Holstein, Arnold-Heller-Str. 3, 24105 Kiel, Germany. anca.remes@uksh.de

[*]Corresponding author: Oliver Müller, Dept. of Internal Med. V, University Hospital Schleswig-Holstein, Arnold-Heller-Str. 3, 24105 Kiel, Germany. oliver.mueller@uksh.de

Supplementary Figures

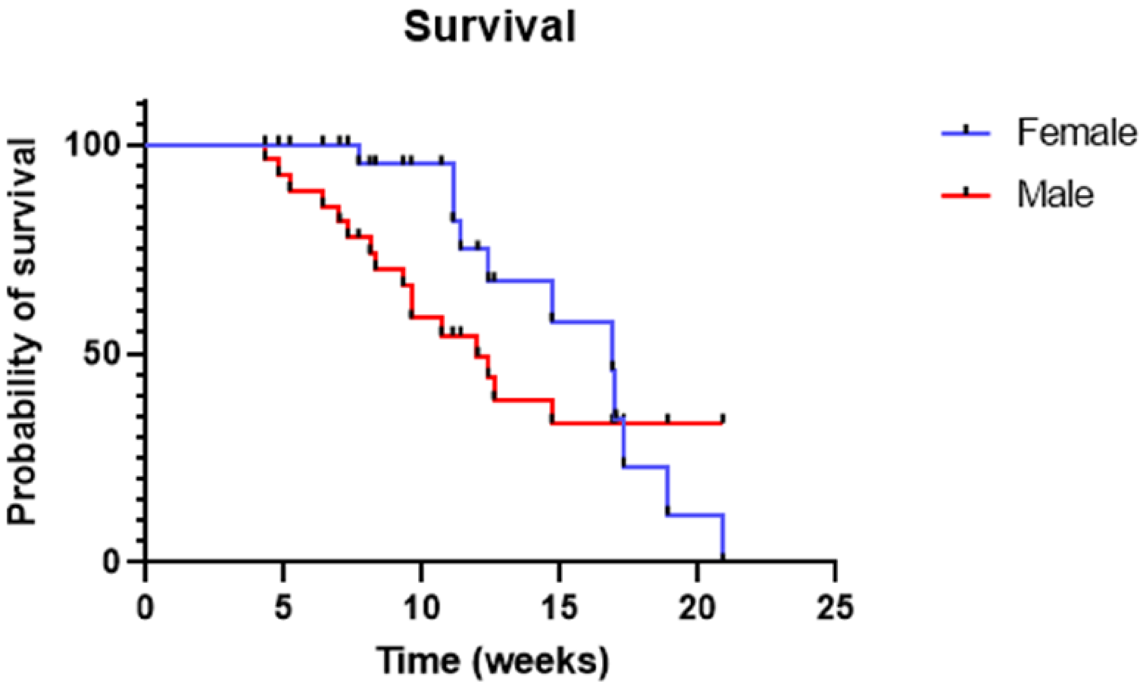

Supplementary Figure 1: Kaplan–Meier survival curve of *mgR/mgR* mice stratified by sex

Cumulative survival probability is plotted over weeks for females (blue) and males (red).

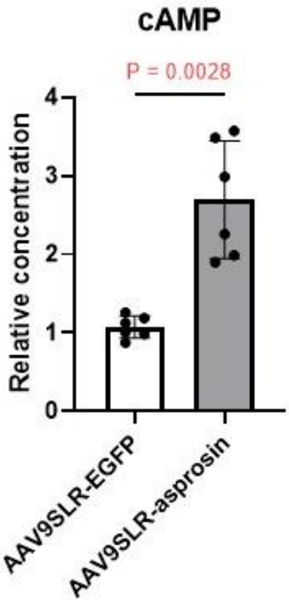

Supplementary Figure 2 Validation of AAV-mediated asprosin bioactivity in HUVECs.

Statistical quantification of cAMP concentration in lysates from HUVEC transduced with AAV9-EGFP or AAV9-asprosin. Data are represented as mean ± SEM

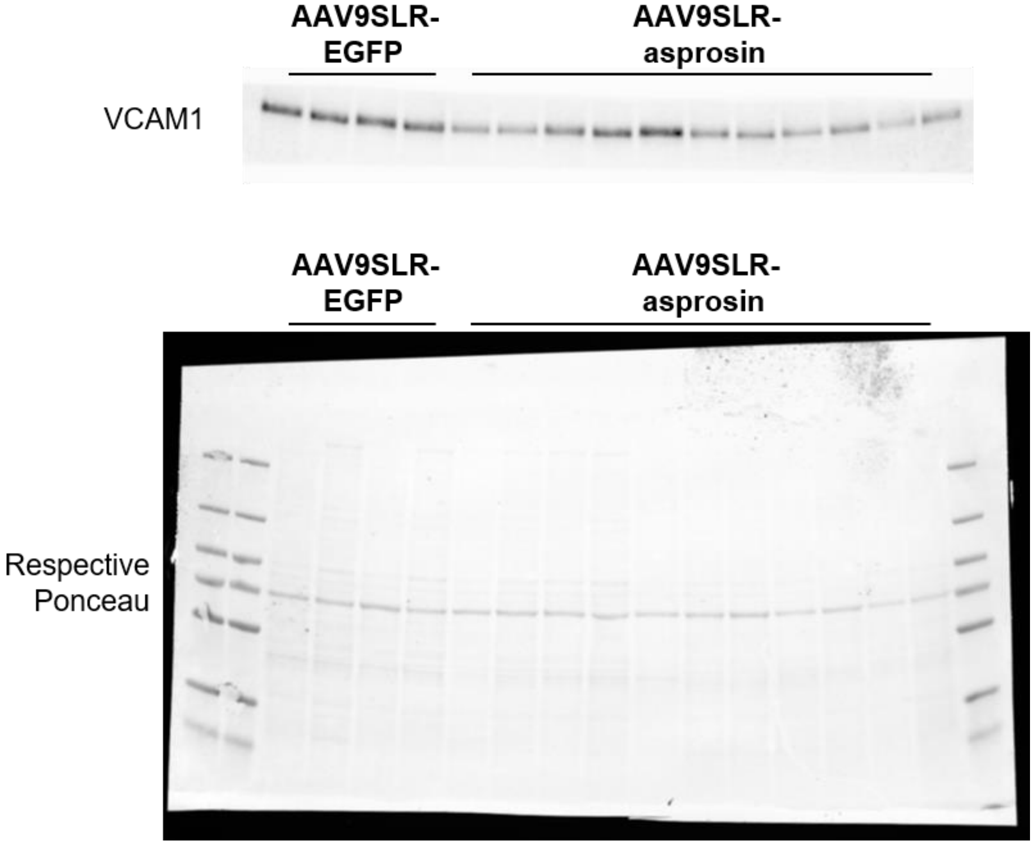

Supplementary Figure 3: Full-length VCAM1 immunoblot corresponding to Fig. 3b, shown together with respective total protein ponceau stain.

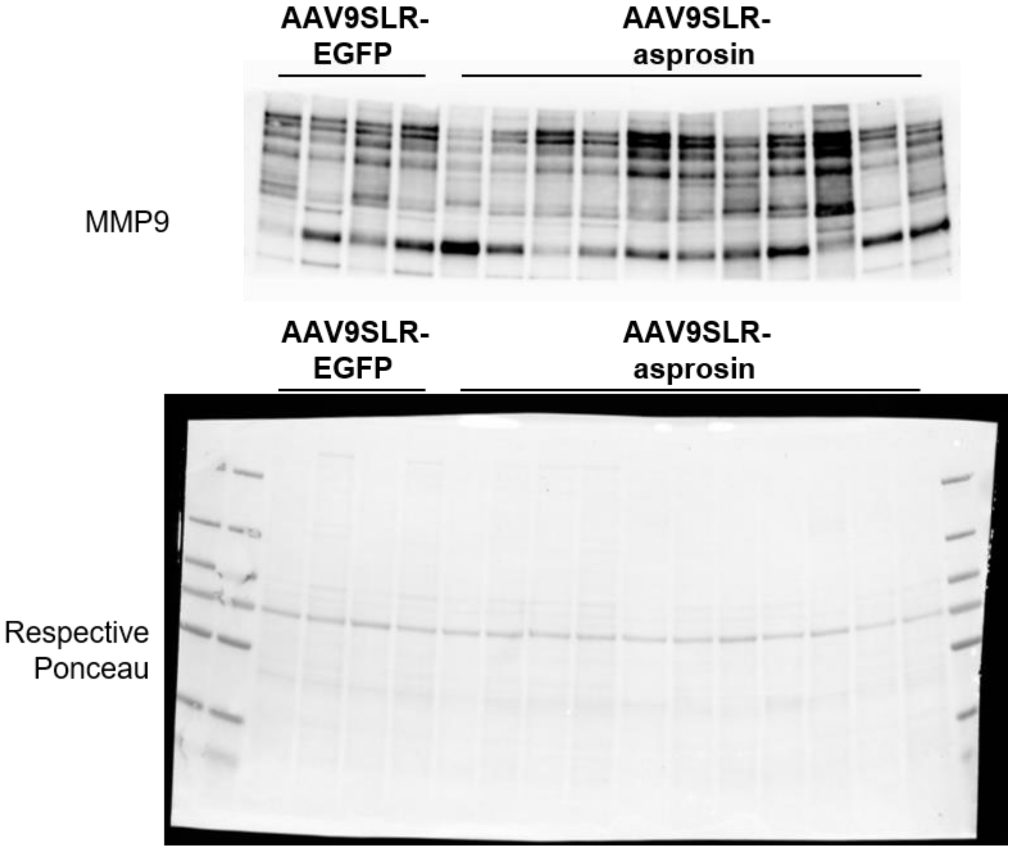

Supplementary Figure 4: Full-length MMP9 immunoblot corresponding to Fig. 3b, shown together with respective total protein ponceau stain.

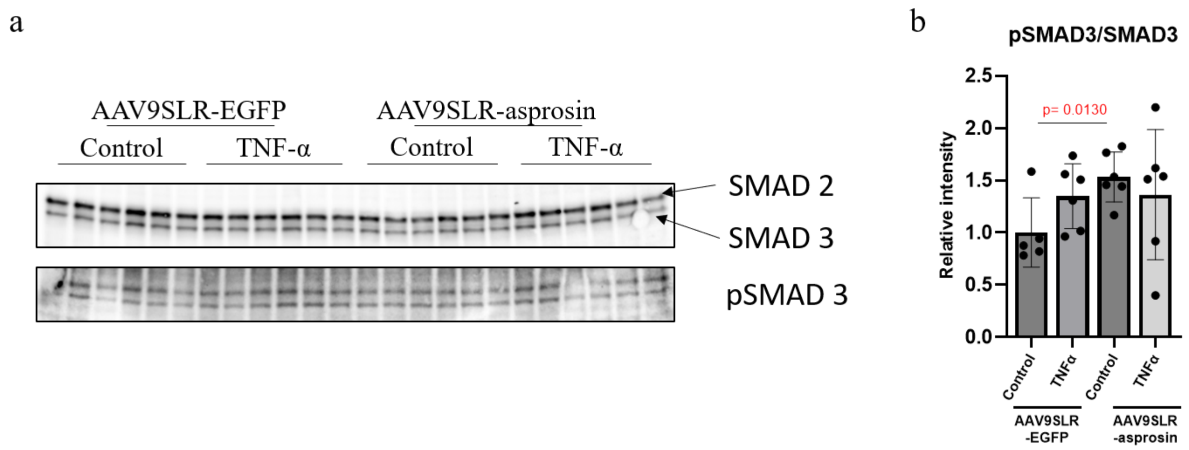

Supplementary Figure 5: (a) SMAD 2/3 and pSMAD3 immunoblot of proteins from MOVAS. (b) Corresponding quantification of ratio of pSMAD3 to SMAD3 protein levels in MOVAS.

Supplementary Table

Supplementary Table S1: Primer List

Gene/Primer name	Sequence
ASPROSIN	F: TCCAGGTTTTTTAAGG
	R: AGCTGCAATAAACAAC
h*IL6*	F: GGTACATCCTCGACGGCATC
	R: TCACCAGGCAAGTCTCCTCA
h*VCAM1*	F: GAATGGGAAGCGAATG
	R: AGATTCACAACTGTGC
h*MMP9*	F: CGAACTTTGAGACAAG
	R: CACTGAGGAAAAGCCC
h*RPLP0*	F: CTGAGCTCCCTGTCTCTCCTCA
	R: CATCTGCTTGGAGCCCACATT
m*Il6*	F: TCCTACCCCAACTTCCAATGCTC
	R: TTGGATGGTCTTGGTCCTTAGCC
m*Vcam1*	F: TGAAGGGATTAGGCTG
	R: CATTTCCACACAGGAG
m*Mmp9*	F: GCGTGTCTGGAGATTCGAC
	R: CCTCATGGTCCACCTTGTTC
AAV-asprosin	F: TCCAGGTTTTTTAAGG
	R: GATACATTGACAAACC
m*Snai1*	F: AAACCCACTCGGATGTGAAG
	R: GAAGGAGTCCTGGCAGTGAG
m*Snai2*	F: CTCACCTCGGGAGCATACAG
	R: GACTTACACGCCCCAAGGATG
m*Rpl32*	F: CTGCTGATGTAAATCT
	R: GCTGTGCTGCTACAAT
